# 晚期NSCLC一线免疫治疗合适的持续时间及其预测因素

**DOI:** 10.3779/j.issn.1009-3419.2026.106.01

**Published:** 2026-01-20

**Authors:** Yue YIN, Xiaotong GUO, Jing AI, Jibo YUE, Lili DENG

**Affiliations:** 150001 哈尔滨，哈尔滨医科大学附属第二医院肿瘤内科; Department of Medical Oncology, Second Affiliated Hospital of Harbin Medical University, Harbin 150001, China

**Keywords:** 肺肿瘤, 免疫治疗, 治疗持续时间, ctDNA, 疗效反应, PD-L1, Lung neoplasms, Immunotherapy, Treatment duration, ctDNA, Efficacy response, PD-L1

## Abstract

晚期非小细胞肺癌（non-small cell lung cancer, NSCLC）的一线免疫治疗在无驱动基因突变患者中显示出显著的生存获益，但其最佳治疗持续时间仍存争议。部分研究支持将免疫治疗限定为2年，认为更长时间的治疗未能带来额外的生存获益；也有研究认为应持续治疗直至疾病进展，以求生存获益最大化。本文系统整理了目前关于免疫治疗持续时间的研究进展，并探讨了基于循环肿瘤DNA（circulating tumor DNA, ctDNA）、最佳疗效反应及程序性死亡配体-1（programmed cell death ligand 1, PD-L1）表达水平等生物标志物在个体化治疗决策中的潜在预测价值。未来仍需更多对比性研究以明确最佳治疗持续时间，并建立基于多维度指标的个体化治疗策略。

## Abstract (en)

First-line immunotherapy for advanced non-small cell lung cancer (NSCLC) shows significant survival benefits in patients without driver mutations, but the optimal duration of treatment remains controversial. Some studies support limiting immunotherapy to 2 years, arguing that longer treatment does not bring additional survival benefits; while other studies believe that treatment should continue until disease progression to maximize survival benefits. This article systematically reviews the current research progress on the duration of immunotherapy and discusses the potential predictive value of biomarkers such as circulating tumor DNA (ctDNA), the best efficacy response, and programmed cell death ligand 1 (PD-L1) expression levels in individualized treatment decisions. More prospective studies, especially biomarker-driven trials, are still needed to clarify the optimal duration of treatment and establish an individualized treatment strategy based on multidimensional indicators.

免疫检查点抑制剂（immune checkpoint inhibitors, ICIs）目前已被确立为无驱动基因突变晚期非小细胞肺癌（non-small cell lung cancer, NSCLC）患者的一线治疗选择，与单纯化疗相比，免疫治疗联合化疗取得更显著的临床结果。然而，目前关于免疫治疗合适的持续时间仍存在诸多不确定因素。KEYNOTE-001^[1]^首次尝试，允许治疗后能维持疾病稳定（stable disease, SD）的患者2年后停止治疗。随后免疫治疗相关研究^[2]^的数据显示，对于SD的晚期肿瘤患者免疫治疗2年后停药仍可以持续获益。但是，仅有不到1/5的患者一线免疫治疗能完成2年，针对这部分人群可以进一步明确固定时长免疫治疗的可行性，并协助探索维持疾病长期稳定的关键因素。另外未来研究或可通过联合用药方案，进一步延长患者一线治疗的无进展生存期（progression-free survival, PFS），但PFS与总生存期（overall survival, OS）之间尚无明确正向关联，因此，不应仅聚焦于一线治疗效果，更应追踪生存预后。值得注意的是，由于尚无明确标志物，仍有研究人员赞同持续免疫治疗直至疾病进展，有研究^[3,4]^发现超过2年的免疫治疗可以为部分患者提供更优的生存结果，所以将最长免疫治疗持续时间定为2年并不是标准化治疗方案。然而，免疫系统过度激活亦可能导致严重不良事件（adverse events, AEs）发生，平衡免疫治疗持续时间与不良事件发生程度亦至关重要^[5]^。

针对一线治疗包含免疫治疗，且最佳缓解率达到SD、完全缓解（complete response, CR）或部分缓解（partial response, PR）（尤其是完成2年治疗且未进展）的晚期NSCLC患者，免疫治疗持续时间应选择固定时间治疗还是持续治疗至疾病进展，目前仍无法确定，2025年中国临床肿瘤学会（Chinese Society of Clinical Oncology, CSCO）指南中亦未对此有明确推荐，需要更多数据支持，以促进实现肿瘤患者个体化治疗。本文旨在总结近年来关于一线免疫治疗持续时间的争论，并针对免疫治疗有效的患者分析其合适的持续时间，探讨相关预测因素，并提出未来研究方向和面临的挑战。

## 1 固定时长的免疫治疗

KEYNOTE、CheckMate等大型临床研究中大部分将一线免疫治疗最长持续时间设定为2年，结果显示，完成35个周期（约2年）免疫治疗的患者生存获益显著。例如，KEYNOTE-189研究^[6]^中57例完成约2年免疫治疗的患者，停药后3年OS率达71.9%，其中23例无疾病进展；类似地，KEYNOTE-407研究^[7]^中亦观察到相近的生存获益，但是二者基线存在差异，鳞状NSCLC患者程序性死亡配体-1（programmed cell death ligand 1, PD-L1）表达水平在1%-49%的人群占比更高，非鳞状NSCLC患者PD-L1表达水平≥50%的人群占比更高，这提示固定2年的免疫治疗可能并非适用于所有患者，仍需继续验证固定时长免疫治疗的可行性，如根据患者肿瘤细胞阳性比例分数（tumor proportion score, TPS）进行更细致的分层分析，并结合肿瘤分型进行亚组分析，比较完成2年免疫治疗与超过2年免疫治疗患者的预后差异。CheckMate 9LA研究^[8]^中，完成2年免疫治疗的54例患者5年OS率和PFS率分别为70%和42%，生存时间大于5年的患者人群中位治疗时间为23.4个月，发现3-4级AEs多发生于免疫治疗的前5个周期，且因AEs停药对疗效无不利影响，提示了固定治疗持续时间的可行性。但仍需探究其他原因停药是否会对疗效产生不利影响，从而确定固定治疗持续时间的适应人群，避免其他患者接受过度治疗。

此外，Sun等^[9]^的研究根据患者免疫治疗持续时间将患者分为固定时间治疗组（≤760 d）和持续治疗组（>760 d），结果显示，两组4年OS率分别为79%和81%，差异无统计学意义[风险比（hazard ratio, HR）= 1.33，95%CI: 0.78-2.25，P=0.29]。该研究提示超过2年的免疫治疗并未明显延长患者的OS，在SD的情况下，治疗满2年后停药并定期随访监测是较为合理的临床策略。Rousseau等^[10]^开展的一项全国性回顾性队列研究同样证实，免疫治疗超过2年与OS改善无关（HR=0.97, 95%CI: 0.75-1.26, P=0.95），该研究对患者的死亡原因进行了更详细的统计分析，免疫治疗持续时间固定为2年的患者中，因肺癌本身死亡者比例更高，这一现象可能与肿瘤逃逸机制密切相关；而免疫治疗持续时间超过2年的患者中，死于非肺癌相关因素者更多，推测与记忆T细胞免疫功能异常、免疫系统过度激活有关。此外，该研究依据患者年龄、免疫治疗线数、所患疾病及使用药物进行更细致的亚组分析，结果显示男性、糖尿病病史、利尿剂使用、β受体阻滞剂使用、止痛药处方史等因素与更差的OS独立关联，其机制可能涉及生活方式、基础疾病和免疫抑制状态，但上述因素是否能直接指导免疫治疗的持续时间，仍需进一步探索。

综上，赞同固定时长的免疫治疗方案，同时也提示长期免疫治疗可以延缓肿瘤进展，但也会升高非肿瘤相关死亡的风险。值得注意的是，各研究在治疗药物选择与亚组设置方面存在差异：Rousseau等^[10]^研究的样本量大，纳入了不同时期使用帕博利珠单抗的患者，主要讨论了各类疾病对OS和治疗持续时间的影响，但未报道组织学类型、PD-L1表达水平和美国东部肿瘤协作组体力状态（Eastern Cooperative Oncology Group performance status, ECOG PS）评分等信息，可能存在一定的选择偏倚。支持固定时间免疫治疗的研究患者基线见表1^[6-9]^。

**表1:** 支持固定时间免疫治疗的研究患者基线

Factors	KEYNOTE-189^[6]^			KEYNOTE-407^[7]^			CheckMate 9LA^[8]^				Sun et al.^[9]^	
	Overall (n=410)	Completed 35 cycles of Pembrolizumab (n=57)		Overall (n=278)	Completed 35 cycles of Pembrolizumab (n=55)		Overall (n=361)	5-yr survivors (n=48)	Completed 35 cycles of immunotherapy (n=52)		760 d fixed duration (n=113)	Fixed duration (n=593)
Treatment line	1			1			1				Any	
Immunotherapy	Pembrolizumab 200 mg q3w			Pembrolizumab 200 mg q3w			Nivolumab q3w+Ipilimumab q6w				Any	
Chemotherapy	Pemetrexed and Carboplatin/Cisplatin			Carboplatin and Paclitaxel/ Nab-paclitaxel			Platinum-based chemotherapy				Any	
Age, yr, median (range)	65 (34-84)	66 (42-82)		65 (29-87)	64 (40-78)		65 (35-81)	64 (42-76)	64 (42-79)		69 (62-75)	69 (62-76)
Histologic type, n (%)												
Squamous	0 (0.0)	0 (0.0)		271 (97.5)	53 (96.4)		115 (31.9)	13 (27.1)	14 (26.9)		29 (25.7)	107 (18.0)
Non-squamous	400 (97.6)	57 (100.0)		7 (2.5)	2 (3.6)		246 (68.1)	35 (72.9)	38 (73.1)		79 (69.9)	463 (78.1)
NOS	10 (2.4)	0 (0.0)		0 (0.0)	0 (0.0)		0 (0.0)	0 (0.0)	0 (0.0)		5 (4.4)	23 (3.9)
Sex, n (%)												
Male	254 (62.0)	34 (59.6)		220 (79.1)	43 (78.2)		252 (69.8)	29 (60.4)	32 (61.5)		51 (45.1)	311 (52.4)
Female	156 (38.0)	23 (40.4)		58 (20.9)	12 (21.8)		109 (30.2)	19 (39.6)	20 (38.5)		62 (54.9)	282 (47.6)
Smoking status, n (%)												
Former or current	362 (88.3)	52 (91.2)		256 (92.1)	52 (94.5)		315 (87.3)	45 (93.8)	50 (96.2)		112 (99.1)	550 (92.7)
Never	48 (11.7)	5 (8.8)		22 (7.9)	3 (5.5)		46 (12.7)	3 (6.2)	2 (3.8)		1 (0.9)	43 (7.3)
ECOG PS, n (%)												
0	185 (45.1)	35 (61.4)		73 (26.3)	18 (32.7)		114 (31.6)	21 (43.8)	17 (32.7)		34 (30.0)	204 (34.3)
1	221 (53.9)	22 (38.6)		205 (73.7)	37 (67.3)		246 (68.1)	27 (56.2)	35 (67.3)		56 (49.6)	254 (42.8)
≥2	1 (0.3)	0 (0.0)		0 (0.0)	0 (0.0)		0 (0.0)	0 (0.0)	0 (0.0)		14 (12.4)	78 (13.3)
Not reported	3 (0.7)	0 (0.0)		0 (0.0)	0 (0.0)		1 (0.3)	0 (0.0)	0 (0.0)		9 (8.0)	57 (9.6)
PD-L1 TPS, n (%)												
Unevaluated	23 (5.6)	3 (5.3)		7 (2.5)	1 (1.8)		157 (43.5)*	23 (47.9)*	19 (36.5)*		27 (23.9)	100 (16.9)
<1%	127 (31.0)	6 (10.5)		95 (34.1)	13 (23.6)						10 (8.8)	90 (15.2)
≥1%	260 (63.4)	48 (84.2)		176 (63.3)	41 (74.5)		204 (56.5)	25 (52.1)	33 (63.5)		76 (67.3)	403 (67.9)
1%-49%	128 (31.2)	17 (29.8)		103 (37.0)	25 (45.5)		128 (35.5)	14 (29.2)	15 (28.8)		25 (22.2)	110 (18.4)
≥50%	132 (32.2)	31 (54.4)		73 (26.3)	16 (29.0)		76 (21.0)	11 (22.9)	18 (34.7)		51 (45.1)	293 (49.5)
Brain metastases, n (%)	73 (17.8)	6 (10.5)		20 (7.2)	2 (3.6)		65 (18.0)	9 (18.8)	13 (25.0)		-	-
Prior therapy, n (%)												
Thoracic radiotherapy	29 (7.1)	5 (8.8)		17 (6.1)	3 (5.5)		-	-	-		-	-
Neoadjuvant therapy	5 (1.2)	0 (0.0)		5 (1.8)	1 (1.8)		-	-	-		-	-
Adjuvant therapy	25 (6.1)	5 (8.8)		-	-		-	-	-		-	-

-: The relevant data is not reported in the cited literature; *The value is the sum of the two data items of PD-L1 TPS unevaluated and <1%. NSCLC: non-small cell lung cancer; NOS: not otherwise specified; ECOG PS: Eastern Cooperative Oncology Group performance status; PD-L1: programmed death-ligand 1; TPS: tumor proportion score.

另有研究指出，不足2年的免疫治疗亦可为患者带来持久的生存获益。Durm等^[11]^的研究发现，PD-L1阳性且接受6个月纳武利尤单抗联合伊匹木单抗治疗的患者OS和PFS与治疗1年的患者相当，出现此结果可能与患者PD-L1表达水平和免疫抑制通路有关：纳武利尤单抗是一种人类免疫球蛋白G4单克隆抗体，可阻断程序性死亡受体-1（programmed cell death protein 1, PD-1）通路；伊匹木单抗为细胞毒性T淋巴细胞相关蛋白4（cytotoxic T-lymphocyte antigen 4, CTLA-4）抑制剂，同时使用可增加抗肿瘤效应。固定时长的免疫治疗可为特定患者带来持久的生存获益，但仍需根据治疗方案等因素综合探讨合适的治疗持续时间，寻找免疫治疗获益与AEs发生风险的平衡点，以指导个体化治疗。

## 2 免疫治疗直至疾病进展

有研究认为长期持续的免疫治疗可进一步延长患者生存期，支持持续免疫治疗直至疾病进展的观点。Cortellini等^[12]^对治疗持续时间进行分析，数据显示不同停药原因对应中位治疗时间分别为4.4、7.5、9.8、24.2、27.7个月，5年OS率分别为7.9%、39.7%、17.6%、73.5%、67.9%。有趣的是无论何种停药原因，治疗至少2年的人群5年OS率均高于整体人群，其中因疾病进展停药和毒性不耐受停药的患者组间差异尤为明显，分别为7.9% vs 31.7%和39.7% vs 72.7%。由此推断，更长时间的免疫治疗可以在一定程度上延长患者OS，尤其对于肿瘤恶性程度高和对ICIs敏感性高的群体，免疫治疗直至疾病进展更适合这部分人群。不足的是该研究并未统计治疗持续时间达到36个月甚至更长时间的患者数据，无法得出治疗超过24个月的患者有生存获益增益。

然而，延长免疫治疗持续时间可以带来更优的生存获益这一结论仍受其他因素干扰，其可靠性需进一步考证：（1）在总体人群中，中位治疗时间为27.7个月对应的5年OS率，低于中位治疗时间为24.2个月对应的5年OS率，这一现象极可能与患者停药原因有关，亦可能由于过长时间的免疫治疗导致生存获益下降，这与Rousseau等^[10]^的研究结论一致。因此，治疗满2年是否为可耐受免疫治疗人群中，生存获益与治疗相关不良反应（treatment-related AEs, TRAEs）之间的临界点，仍需进一步探索验证；（2）研究基础人群存在差异，治疗至少2年的人群较总体人群而言，已天然排除肿瘤恶性程度高、疾病进展快的患者，将该部分人群与整体人群相比，不可避免地存在混杂偏倚，从而可能影响结论的真实性。

Borghaei等^[13]^汇总了CheckMate 017和CheckMate 057的数据，发现完成2、3、4年纳武利尤单抗治疗且SD的患者1年PFS率分别为76.1%、89.5%、87.5%；5年PFS率分别为59.6%、78.3%、87.5%；5年OS率分别为82.0%、93.0%、100%，生存时间超过5年的患者人群中位治疗时间为36.9个月，大于CheckMate 9LA中的23.4个月，且未发生5级AEs，未发生额外安全风险。Geier等^[14]^在研究中同样支持超过2年免疫治疗可以改善生存，且未明显增加TRAEs发生率。上述两项研究主要针对先前经过铂类治疗后进展的NSCLC患者，是否适用于一线免疫治疗还需进一步明确，但可以明确的是，纳武利尤单抗的治疗持续时间对其安全性影响极小。

Borghaei等^[13]^的研究与CheckMate 9LA^[8]^中免疫治疗的中位治疗时间存在差异，其原因可能是：（1）治疗药物不同，两项研究虽均包含纳武利尤单抗，但CheckMate 9LA中采用联合伊匹木单抗和化疗的方案，可同时阻断PD-1通路和CTLA-4，在抑制T细胞对肿瘤的免疫监视的同时增强了T细胞的活化和增殖，相较于Borghaei等^[13]^的研究，整体抗肿瘤作用更强；（2）治疗线数不同，Borghaei等^[13]^的研究为二线治疗，而临床数据提示早期治疗通常可带来更优的生存获益，据此推测，二线及后线治疗可能需要更长的治疗持续时间，但目前尚无直接证据支持此观点，仍需进一步验证。

CheckMate 153研究^[15]^中发现持续治疗组中位PFS显著优于1年固定时间组（24.7 vs 9.4个月，HR=0.56，95%CI: 0.37-0.84），且在各种子集中持续治疗组均有更长的中位PFS；持续治疗组中位OS亦显著优于1年固定时间组（未达到 vs 28.8个月，HR=0.62，95%CI: 0.42-0.92），提示1年的免疫治疗可能不足，需延长甚至持续治疗直至疾病进展。与Checkmate 9LA^[8]^相比，该研究中未联合化疗且治疗线数不同，可能影响机体对ICIs的敏感性。另外，Huang等^[16]^应用拟合方法发现OS增益与免疫治疗持续时间有关，2年免疫治疗较1年免疫治疗有更好的OS，且OS增益与肿瘤组织学、患者PS评分、放疗剂量、免疫治疗药物类型或免疫治疗开始时间无关联。支持免疫治疗直至疾病进展的研究患者基线见表2^[13-15]^。

**表2:** 支持免疫治疗直至疾病进展的研究患者基线

Factors	Borghaei et al.^[13]^			Geier et al.^[14]^					CheckMate 153^[15]^			
	<1-yr survivors (n=222)	≥5-yr survivors (n=50)		Long-term survivors (n=65)	<2-yr Nivolumab (n=47)	2-yr Nivolumab (n=7)	>2-yr Nivolmab (n=11)		PFS population 1-yr fixed duration (n=85)	PFS population indefinite duration (n=89)	ITT population 1-yr fixed duration (n=125)	ITT population indefinite duration (n=127)
Treatment line	≥2			≥2					Any			
Immunotherapy	Nivolumab 3 mg/kg q2w			Nivolumab 3 mg/kg q15d					Nivolumab 3 mg/kg q2w			
Chemotherapy	No			No					No			
Age, yr, median (range)	62 (37-85)	60 (41-74)		59 (39-80)	59 (45-80)	52 (39-72)	58 (53-66)		67 (49-86)	66 (34-92)	67 (49-86)	66 (34-92)
Histologic type, n (%)												
Squamous	77 (34.7)	14 (28.0)		15 (23.1)	11 (23.4)	3 (42.9)	1 (9.1))		35 (41.2)	28 (31.5)	55 (44.0)	34 (26.8)
Non-squamous	145 (65.3)	36 (72.0)		48 (73.8)	35 (74.5)	4 (57.1)	9 (81.8)		50 (58.8)	61 (68.5)	70 (56.0)	93 (73.2)
NOS	0 (0.0)	0 (0.0)		2 (3.1)	1 (2.1)	0 (0.0)	1 (9.1)		0 (0.0)	0 (0.0)	0 (0.0)	0 (0.0)
Sex, n (%)												
Male	140 (63.1)	31 (62.0)		48 (73.8)	35 (74.5)	5 (71.4)	8 (72.7)		45 (52.9)	47 (52.8)	63 (50.4)	60 (47.2)
Female	82 (36.9)	19 (38.0)		17 (26.2)	12 (25.5)	2 (28.6)	3 (27.3)		40 (47.1)	42 (47.2)	62 (49.6)	67 (52.8)
Smoking status, n (%)												
Former or current	185 (83.3)	42 (84.0)		57 (87.7)	39 (83.0)	7 (100.0)	11 (100.0)		82 (96.5)	83 (93.3)	115 (92.0)	114 (89.8)
Never	33 (14.9)	6 (12.0)		8 (12.3)	8 (17.0)	0 (0.0)	0 (0.0)		3 (3.5)	6 (6.7)	10 (8.0)	13 (10.2)
Not reported	4 (1.8)	2 (4.0)		0 (0.0)	0 (0.0)	0 (0.0)	0 (0.0)		0 (0.0)	0 (0.0)	0 (0.0)	0 (0.0)
ECOG PS, n (%)												
0	37 (16.7)	20 (40.0)		-	-	-	-		33 (38.8)	32 (36.0)	45 (36.0)	40 (31.5)
1	183 (82.4)	30 (60.0)		-	-	-	-		50 (58.8)	51 (57.3)	78 (62.4)	80 (63.0)
≥2	0 (0.0)	0 (0.0)		-	-	-	-		2 (2.4)	6 (6.7)	2 (1.6)	7 (5.5)
Not reported	2 (0.9)	0 (0.0)		-	-	-	-		0 (0.0)	0 (0.0)	0 (0.0)	0 (0.0)
Clinical stage, n (%)												
Stage IIIB	25 (11.3)	9 (18.0)		-	-	-	-		10 (11.8)	11 (12.4)	12 (9.6)	12 (9.4)
Stage IV	197 (88.7)	41 (82.0)		-	-	-	-		75 (88.2)	78 (87.6)	113 (90.4)	115 (90.6)
EGFR mutation status, n (%)												
Positive	26 (11.7)	2 (4.0)		1 (1.5)	1 (2.1)	0 (0.0)	0 (0.0)		-	-	-	-
Not detected	78 (35.1)	23 (46.0)		64 (98.5)*^a^	46 (97.9)*^a^	7 (100.0)*^a^	11 (100.0)*^a^		-	-	-	-
Not reported	118 (53.2)	25 (50.0)							-	-	-	-
ALK mutation status, n (%)												
Positive	3 (1.3)	1 (2.0)		-	-	-	-		-	-	-	-
Not detected	59 (26.6)	14 (28.0)		-	-	-	-		-	-	-	-
Not reported	160 (72.1)	35 (70.0)		-	-	-	-		-	-	-	-
PD-L1 TPS, n (%)												
Evaluable	176 (79.3)	40 (80.0)		-	-	-	-		50 (58.8)	50 (56.2)	61 (48.8)	65 (51.2)
<1%	90 (40.6)	10 (20.0)		-	-	-	-		12 (14.1)	20 (22.5)	16 (12.8)	24 (18.9)
≥1%	86 (38.7)	30 (60.0)		-	-	-	-		38 (44.7)	30 (33.7)	45 (36.0)	41 (32.3)
≥10%	47 (26.7)	26 (65.0)		-	-	-	-		-	-	-	-
≥50%	27 (15.3)	22 (55.0)		-	-	-	-		12 (14.1)	12 (13.5)	14 (11.2)	18 (14.2)
Unevaluable	46 (20.7)*^b^	10 (20.0)*^b^		-	-	-	-		6 (7.1)	12 (13.5)	12 (9.6)	18 (14.2)
Not reported				-	-	-	-		29 (34.1)	27 (30.3)	52 (41.6)	44 (34.6)
Best therapeutic response, n (%)												
CR	-	5 (10.0)		7 (10.8)	5 (10.6)	1 (14.3)	1 (9.1)		58 (68.2)*^c^	62 (69.7)*^c^	58 (46.4)*^c^	62 (48.8)*^c^
PR	-	34 (68.0)		33 (50.8)	19 (40.4)	5 (71.4)	9 (81.8)					
SD	-	8 (16.0)		21 (32.3)	19 (40.4)	1 (14.3)	1 (9.1)		27 (31.8)	27 (30.3)	27 (21.6)	27 (21.3)
PD	-	3 (6.0)		4 (6.2)	4 (8.5)	0 (0.0)	0 (0.0)		0 (0.0)	0 (0.0)	40 (32.0)	38 (29.9)

-: The relevant data is not reported in the cited literature; *^a^The value is the sum of the two data items of EGFR mutation status not detected and not reported; *^b^The value is the sum of the two data items of PD-L1 status unevaluable and not reported; *^c^The value is the sum of the two data items of best therapeutic response CR and PR. ITT population: all patients who continued to receive treatment at 1 year and were randomly assigned, regardless of response status; PFS population: patients who did not have PD and had not initiated other systemic anticancer therapy before random assignment. ITT: intention-to-treat; PFS: progression-free survival; EGFR: epidermal growth factor receptor; ALK: anaplastic lymphoma kinase; CR: complete response; PR: partial response; SD: stable disease; PD: progressive disease.

此外多项研究^[17,18]^显示，免疫治疗停止后若出现疾病进展，再次使用原免疫药物仍可取得疾病控制。KEYNOTE-024中完成35个周期免疫治疗后发生疾病进展的14例患者，12例再次接受了帕博利珠单抗治疗，其中4例达到PR，6例SD，由此推断长期不间断的帕博利珠单抗治疗可能增加患者的生存获益^[19]^。这些结果均提示长期不间断的免疫治疗可能带来更佳的生存结果，但是否存在免疫治疗获益“平台期”仍未可知，验证此猜想仍需继续建立对比试验。

## 3 免疫治疗持续时间的争议与讨论

现有研究并未明确免疫治疗持续时间与患者生存获益之间的关联，且长期免疫治疗可能增加非肿瘤相关死亡风险，这也导致学界对免疫治疗持续时间的观点并不统一。各项研究结论见表3^[6-10,13-15]^，TRAEs发生率见表4^[6-8,13-15]^。这一现状的成因可能与目标人群的筛选偏差有关，包括治疗方案选择差异、目标人群个体差异和临床治疗效果差异。因此，亟须搭建免疫治疗持续时间与生存获益间的关系桥梁，明确二者内在联系。鉴于治疗效果与免疫治疗持续时间和OS密切关联，临床中可尝试基于患者免疫检查点差异及最佳疗效反应，构建预测模型，以实现对免疫治疗持续时间的精准预判。

**表3:** 各研究结论

Study	Group	ORR, n (%)	mDoR (mon)	mPFS (mon)	mOS (mon)		PFS rate (%)						OS rate (%)					
							1-yr	2-yr	3-yr	4-yr	5-yr		1-yr	2-yr	3-yr	4-yr	5-yr	6-yr
KEYNOTE-189^[6]^	Completed 35 cycles of Pembrolizumab (n=57)	49 (86.0)	57.7 (range: 4.2-68.3+)	NR (95%CI: 14.7-NR)	-		-	-	-	-	56.2 (95%CI: 39.7-69.8)		-	-	-	-	71.9 (95%CI: 58.3-81.8)	-
KEYNOTE-407^[7]^	Completed 35 cycles of Pembrolizumab (n=55)	50 (90.9)	NR (range: 7.1-61.5+)	NR (95%CI: 21.2-NR)	-		-	-	-	-	58.4 (95%CI: 39.8-73.0)		-	-	-	-	69.5 (95%CI: 54.8-80.2)	-
CheckMate 9LA^[8]^	Completed 35 cycles of immunotherapy (n=52)	40 (76.9)	NR (95%CI: 33.6-NR)	44.8 (95%CI: 34.6-NR)	NR (95%CI: 63.6-NR)		-	-	-	-	42 (95%CI: 28.0-56.0)		-	-	-	-	70 (95%CI: 46.0-81.0)	-
Sun et al.^[9]^	760-day fixed duration (n=113)	-	-	-	-		-	-	-	-	-		-	-	89 (95%CI: 81.0- 94.0)	79 (95%CI: 66.0- 87.0)	-	-
	Fixed duration (n=593)	-	-	-	-		-	-	-	-	-		-	-	91 (95%CI: 88.0- 94.0)	81 (95%CI: 77.0- 85.0)	-	-
Rousseau et al.^[10]^	29-mon fixed duration (n=919)	-	-	-	-		-	-	-	-	-		-	-	-	96.1 (95%CI: 94.7- 97.5)	87.3 (95%CI: 84.4- 90.3)	77.2 (95%CI: 72.2- 82.6)
	Indefinite duration (n=2165)	-	-	-	-		-	-	-	-	-		-	-	-	95.0 (95%CI: 94.0- 96.0)	85.6 (95%CI: 83.7- 87.6)	77.0 (95%CI: 74.2- 80.0)
Borghaei et al.^[13]^	2-yr Nivolumab (n=45)	-	-	-	-		-	100.0	76.1	68.2	59.6		-	-	-	-	82.0	-
	3-yr Nivolumab (n=29)	-	-	-	-		-	-	100.0	89.5	78.3		-	-	-	-	93.0	-
	4-yr Nivolumab (n=25)	-	-	-	-		-	-	-	100.0	87.5		-	-	-	-	100.0	-
Geier et al.^[14]^	Long-term survivors (n=65)	40 (61.5)	-	28.4 (95%CI: 21.4-NR)	NR (95%CI: 32.6-NR)		-	-	37.8	-	-		-	-	62.1	-	-	-
	<2-yr Nivolumab (n=47)	24 (51.0)	-	20.0 (95%CI: 11.3-28.2)	32.7 (95%CI: 29.7-NR)		-	44.7	30.4	-	-		-	-	49.0	-	-	-
	2-yr Nivolumab (n=7)	6 (85.7)	-	NR	NR		-	100.0	85.7	-	-		-	-	85.7	-	-	-
	>2-yr Nivolumab (n=11)	10 (90.9)	-	NR	NR		-	100.0	72.0	-	-		-	-	100.0	-	-	-
CheckMate 153^[15]^	PFS population 1-yr fixed duration (n=85)	58 (68.2)	-	9.4 (95%CI: 5.6-13.0)	32.5 (95%CI: 22.9-NR)		44.0	30.7	-	-	-		82.0	60.9	-	-	-	-
	PFS population indefinite duration (n=89)	62 (68.7)	-	24.7 (95%CI: 14.8-34.7)	NR (95%CI: 33.6-NR)		64.6	51.9	-	-	-		86.1	73.4	-	-	-	-

.==-: The relevant data is not reported in the cited literature. ORR: objective response rate; mDoR: median duration of response; mPFS: median progression-free survival; mOS: median overall survival; NR: not reached.

**表4:** 各研究治疗相关不良反应发生率

Study	Group	TRAEs, n (%)	Grade 3-4 TRAEs, n (%)	≥grade 3 irAEs, n (%)
KEYNOTE-189^[6]^	Completed 35 cycles of Pembrolizumab (n=57)	57 (100.0)	38 (66.7)	7 (12.3)
KEYNOTE-407^[7]^	Completed 35 cycles of Pembrolizumab (n=55)	55 (100.0)	35 (63.6)	1 (1.8)
CheckMate 9LA^[8]^	Overall (n=358)	328 (91.6)	5 (1.4)	-
	≥5-yr survivors and 1-5 doses of Ipilimumab (n=12)	12 (100.0)	11 (91.7)	7 (58.3)
	≥5-yr survivors and 6-15 doses of Ipilimumab (n=12)	12 (100.0)	7 (58.3)	5 (41.7)
	≥5-yr survivors and 16-18 doses of Ipilimumab (n=24)	23 (95.8)	11 (45.8)	1 (4.2)
Borghaei et al.^[13]^	Overall (n=418)	284 (67.9)	45 (10.8)	-
	3-5 years' follow-up (n=31)	8 (25.8)	1 (3.2)	-
Geier et al.^[14]^	Long-term survivors (n=65)	44 (67.7)	17 (26.2)	-
	<2-yr Nivolumab (n=47)	30 (63.8)	16 (34.0)	-
	2-yr Nivolumab (n=7)	6 (85.7)	0 (0.0)	-
	>2-yr Nivolumab (n=11)	8 (72.7)	1 (9.1)	-
CheckMate 153^[15]^	ITT population 1-yr fixed duration (n=125)	33 (26.4)	40 (32.0)	-
	ITT population indefinite duration (n=127)	61 (48.0)	12 (9.4)	-

-: The relevant data is not reported in the cited literature; ITT population: all patients who continued to receive treatment at 1 year and were randomly assigned, regardless of response status. TRAEs: treatment-related adverse events; irAEs: immune-related adverse reactions.

## 4 特殊人群免疫治疗讨论

在老年肿瘤人群中免疫治疗较化疗更具有安全性，因此更倾向于选择免疫治疗。免疫治疗疗效依赖于T细胞活化与增殖，而免疫功能随年龄的增长而下降，其最显著特征为胸腺萎缩，亦可理解为稳定的增殖停滞，对有丝分裂信号无应答^[20,21]^。多种标志物（如端粒缩短、线粒体功能障碍、细胞传导通路损伤等）的协同作用可共同推进免疫衰老^[22,23]^。因此，免疫治疗在老年人群中的持久性仍有待商榷。以小鼠为研究对象的实验^[24-26]^中发现，亚精胺、树突状细胞超激活剂、抗原特异性CD8^+^ T细胞等均具有抗免疫衰老的潜力，有望在未来为老年肿瘤患者提供新的治疗策略。另有研究^[27]^表明，老年肿瘤患者更易出现免疫相关AEs（immune-related AEs, irAEs），而白细胞介素-6受体阻断剂与ICIs相结合，既可以降低发生irAEs风险，也可以增加抗肿瘤效应，但体内白细胞介素-6受体水平与irAEs缓解速度并无关系，且目前亦并不清楚其内在机制。

在合并自身免疫性疾病的肿瘤人群中，ICIs会加重AEs^[28]^，欧洲肿瘤内科学会（European Society for Medical Oncology, ESMO）临床实践指南^[29]^表明细胞因子抑制剂可作为治疗难治性irAEs的预防策略，但是针对不同AEs，其具体机制尚未明确。在Gohil等^[30]^研究中显示，针对合并自身免疫性疾病的肿瘤人群，ICIs仍能提高生存期，且从数据中发现治疗肿瘤前未接受过免疫抑制剂治疗的人群，耐受ICIs时间更长，虽然这可能与自身免疫性疾病的严重程度有关，但也能进一步提示联合用药可以增加机体对ICIs的耐受程度。

针对特殊人群的抗肿瘤治疗方案中ICIs必不可少，多药物联合使用以增加ICIs使用时间可能是未来的发展方向。深入解析T细胞内在调节机制、宿主代谢及肿瘤微环境改变迫在眉睫，不仅有助于进一步阐明抗肿瘤药物在特殊人群中的作用机制，还可为设计合理的联合用药策略、实现个体化治疗提供理论依据和新思路。

## 5 预测免疫治疗持续时间的相关因素

### 5.1 循环肿瘤DNA（circulating tumor DNA, ctDNA）动态监测

近年来ctDNA在预测肿瘤进展与治疗效果等方面显示出重要价值。研究^[31]^发现，肿瘤可将ctDNA释放到外周血中，即血浆游离DNA（cell-free DNA, cfDNA），使ctDNA得以检测和量化，且ctDNA动态变化（通过变异等位基因分数的平均值及cfDNA浓度计算）与免疫治疗疗效密切关联，ctDNA水平下降提示肿瘤消退治疗反应良好，反之则提示疾病进展，肿瘤免疫逃逸机制激活。因此，通过ctDNA指导免疫治疗持续时间是未来可行方案之一。Fitzgerald等^[32]^指出，接受免疫治疗后绝大部分患者发生ctDNA浓度改变，且ctDNA浓度与病情演变一致。其他研究^[33,34]^亦证实，免疫治疗后ctDNA水平降低和ctDNA阴性（治疗开始前和治疗后均未检测出ctDNA）与更优的OS、PFS有关联，通过ctDNA的动态变化预测免疫治疗的停药时间似乎可行。

Moding等^[35]^分析经放化疗好转后接受巩固期免疫治疗的患者数据，发现对于放化疗后ctDNA阴性患者，无论是否接受巩固期免疫治疗均能获得良好预后，提示ctDNA阴性患者无需接受额外的免疫治疗；对于放化疗后ctDNA阳性患者，可以根据巩固期免疫治疗期间ctDNA水平变化决定是否继续接受免疫治疗。巩固期免疫治疗期间ctDNA水平升高者，继续接受免疫治疗获益甚微，巩固期免疫治疗期间ctDNA水平下降者继续接受免疫治疗能提高患者预后。Wang等^[36]^认为放化疗后ctDNA阳性的患者巩固期ICIs治疗显著提高了OS，在疾病进展前ctDNA水平升高，并发现血肿瘤突变负荷（blood tumor mutational burden, bTMB）变化可以协助预测ctDNA阳性患者预后。由于该研究采集患者血浆时机仅在放化疗前、放化疗后和疾病进展前，因此未涉及ctDNA水平变化分析。综合以上分析，治疗后ctDNA阴性患者建议停止免疫治疗；治疗后ctDNA阳性患者需继续免疫治疗并密切观察ctDNA动态变化（如每3个月测量1次ctDNA浓度），再结合bTMB等指标决定是否终止免疫治疗。

尽管ctDNA动态监测有望指导停药决策，但其动态变化具有复杂性、灵敏度检测难度大且技术平台有限等特点。Janke等^[37]^收集健康人群和NSCLC人群的外周静脉血，用拷贝数异常分数评分表示染色体不稳定程度，发现NSCLC人群的血浆样本以拷贝数变异和cfDNA碎片特征为特点，且二者为互补指标，联合分析可有效提高ctDNA检测率^[38]^。但目前ctDNA相关分析尚未形成统一、简便的标准化计算方法，仍需进一步完善。

### 5.2 最佳疗效反应

最佳疗效反应为治疗效果的回归性指标，可以间接反映机体对治疗的敏感程度，Salomone等^[39]^揭示客观缓解率（objective response rate, ORR）与OS存在中等联系，根据治疗方案不同，PFS与OS之间亦存在不同程度联系，由此推测ICIs的抗肿瘤疗效可能存在时效性，免疫治疗持续时间或可基于治疗后的最佳疗效进行个体化评估。此外，Mahiat等^[40]^的研究也证实，完成2年免疫治疗的晚期NSCLC患者，PFS似乎与放射学反应有关，但是该研究并未深究其与OS的关系，以及继续治疗对预后的影响。

回顾既往研究^[14]^发现达到PR/SD的患者更易发生疾病进展。CheckMate 153研究^[15]^显示，达到CR/PR的患者中持续治疗相比于1年固定时间治疗有更好的中位PFS（31.0 vs 10.6个月，HR=0.46，95%CI: 0.27-0.77）和OS（未达到 vs 33.5个月，HR=0.50，95%CI: 0.26-0.97），而在SD患者中两组中位PFS无显著差异（11.8 vs 9.4个月，HR=1.01，95%CI: 0.51-2.01）且OS相似（32.2 vs 26.6个月，HR=0.88，95%CI: 0.42-1.84），提示免疫治疗1年后达到CR/PR的患者继续治疗似乎有更好的预后。然而，Ding等^[41]^的研究中，亚组分析显示达到CR/PR的患者，持续治疗组（治疗持续时间≥18个月）和固定治疗组（治疗持续时间<18个月）的中位OS无统计学差异，而达到SD的患者，持续治疗组的中位OS高于固定治疗组（P=0.019），提示在治疗18个月后达到SD的人群更倾向于继续接受免疫治疗。这是否意味着，对于达到CR/PR的患者，固定1年的治疗持续时间可能不足，需将治疗延长至18个月；而对于SD的患者，即使治疗18个月仍可能不足，其最佳治疗持续时间仍有待进一步探索。以上这种差异可能反映了不同研究人群、治疗方案（如是否联合化疗）及治疗持续时间本身对疗效反应的复杂影响。Ding等^[41]^的研究提示，对于SD患者，更长的治疗时间（≥18个月）可能带来生存获益，这或许与免疫反应的累积效应或患者筛选有关。

综合来看，1年免疫治疗对于晚期NSCLC患者获益有限，长期免疫治疗可能是更优的选择。对于达到CR/PR的患者，可考虑固定时长的免疫治疗，但仍需要更多的数据验证合适的时间，而对于SD患者，则建议持续治疗超过24个月甚至治疗至疾病进展。

### 5.3 PD-L1表达水平

PD-L1抑制剂通过阻止PD-1与PD-L1偶联，从而起到抗肿瘤的作用。机体对PD-1/L1抑制剂敏感性不同，肿瘤PD-L1表达水平亦不同，通过PD-L1的表达水平预测免疫治疗持续时间可能成为未来研究方向之一。目前PD-L1检测的金标准为免疫组织化学（immunohistochemistry, IHC）法，TPS评估其表达水平。OAK研究^[42]^结果显示长期生存组（≥2年）PD-L1的表达水平和T细胞效应基因信号表达更高，提示该群体可能从免疫治疗中得到更多获益。但该研究目标人群先前经过1-2次化疗，不排除表皮生长因子受体（epidermal growth factor receptor, EGFR）或间变性淋巴瘤激酶（anaplastic lymphoma kinase, ALK）融合基因阳性人群，且长期生存人群包括治疗后进展人群，可能导致结果过于笼统。CameL研究^[43]^排除了EGFR或ALK基因阳性患者，发现PD-L1高表达的人群更易完成长时间的免疫治疗且5年OS率更高。KEYNOTE-042^[44]^和KEYNOTE-010^[45]^横向对比可见，KEYNOTE-042中帕博利珠单抗中位治疗时间更长（6.5 vs 3.5个月），中位OS更长（20.2 vs 11.8个月），5年OS率更高（19.0% vs 15.6%），提示在TPS≥1%的人群中，更长疗程的免疫治疗可能更具优势。然而，在完成2年免疫治疗的患者中KEYNOTE-010的5年OS率反而更高（83.0% vs 56.6%），导致这一差异可能与患者基线特征有关：KEYNOTE-010未排除EGFR或ALK阳性患者，且该部分患者接受了酪氨酸酶抑制剂治疗。Gadgeel等^[46]^的研究显示，TPS<1%且完成2年免疫治疗的人群5年OS率为56.7%，与KEYNOTE-042研究^[44]^结果相似，但该研究纳入全球范围内的晚期NSCLC患者，研究人群存在一定差异。

另外，多项研究^[47-49]^报道，无论PD-L1表达如何，重启免疫治疗仍能控制疾病进展。KEYNOTE-010研究^[45]^中接受第二阶段帕博利珠单抗治疗的人群中TPS<50%人群占比较完成第一阶段帕博利珠单抗治疗的人群中TPS<50%人群占比多，提示TPS<50%的人群发生疾病进展可能性更高，可能更适合接受长期免疫治疗。但该结论基于再治疗数据，存在选择偏倚。除此之外，这是否意味着免疫治疗存在获益“平台期”，且PD-L1低表达的人群“平台期”更短。未来还需更多研究验证对免疫治疗“平台期”的猜想。

值得注意的是，PD-L1表达并非一成不变，其在治疗过程中可能存在时空异质性^[50]^。Takahashi^[51]^的个案分析中，通过Aperio ImageScope评估患者PD-L1表达水平，发现帕博利珠单抗治疗前后PD-L1表达不同，经过长时间的免疫治疗其表达可能下调。此外，PD-L1检测方法种类繁多，易导致TPS分层存在偏差。例如，IHC检测平台不统一，OAK研究^[52]^采用VENTANA SP142平台评估PD-L1表达水平，而KEYNOTE-010研究^[45]^则使用DAKO 22C3平台评估，且在回顾性研究中无法保证所有研究对象均采用同一种IHC平台检测PD-L1表达。因此，未来研究中还需统一PD-L1检测方法，减少选择偏倚，提高结果的可靠性与可比性。

以上研究结论亦为我们提供了新的思路，是否可以利用机体对PD-1/L1抑制剂的敏感性和PD-L1表达水平进行综合评估，根据评估结果决定免疫治疗持续时间和重启免疫治疗的时机，如评估结果为阳性人群则缩短免疫治疗持续时间，延长停药时长；评估结果为阴性人群则延长免疫治疗持续时间，缩短停药时长。

### 5.4 其他预测因素

ICIs的抗肿瘤作用主要依赖T细胞激活效应，目前发现肿瘤基因组特征（包括肿瘤表面PD-L1表达、特定基因突变和肿瘤微环境）和信号传导通路改变均会影响T细胞抗肿瘤效应，破解T细胞功能调控的内在机制，可为免疫治疗停药标准制定提供关键指导。除以上因素，亦有研究^[53,54]^发现其他潜在生物标志物，涵盖治疗相关因素（如irAEs发生情况、疾病缓解持续时间等）与宿主因素（如肠道微生物菌群、代谢状态、染色体稳定程度、耐药基因等）。但目前有关的研究样本量有限，证据等级偏低，其临床应用价值仍需更多高质量研究进一步验证。

最后需重点强调，预测免疫治疗持续时间绝非单一因素分析可实现，需整合ctDNA动态变化、临床疗效分层、PD-L1表达水平等多方面开展多维度分析，以构建更系统、全面的预测模型框架，为临床决策提供科学依据。

## 6 总结与挑战

目前能实现长期SD的患者相关数据匮乏，尤其是能够维持2年以上稳定状态的患者数据。针对免疫治疗合适时间的研究不仅数量有限，且多以回顾性队列分析为主，为弥补亚组分型领域的研究不足，未来应开展更多高质量的随机对照试验，重点关注特殊人群，如年龄≥75周岁、合并肝肾功能损害及既往患有自身免疫疾病的患者。此类人群因生理机能存在特殊性，在治疗决策中需更加注重平衡免疫治疗获益和irAEs风险，更应以个体化评估为核心，根据个体差异制定适合的治疗持续时间并加以动态调整；同时，建议设计多因素对照实验，例如开展以“ctDNA动态变化+疗效反应+PD-L1表达”为联合预测指标的随机对照试验，将OS设为主要研究终点，PFS和AEs率设为次要研究重点。待有足够的循证医学证据支持后，开展前瞻性研究并建立整合性预测模型，以推动肿瘤患者个体化治疗的发展及实行。

由于患者之间的个体化差异，探寻免疫治疗的最佳持续时间颇具挑战性。现阶段长期的免疫治疗仍是临床中的首要选择，然而由于患者基线（性别、基础疾病、PD-L1表达水平、基因突变等）和治疗方案（药物种类、治疗线数等）均会影响OS，致使临床专家难以精确定位合适的免疫治疗持续时间。此外，除疾病本身因素外，仍需多维度考虑经济条件、医院规模等外在因素对免疫治疗持续时间的潜在影响。未来还需结合患者治疗后的ECOG PS、疗效分层、AEs类型、影像学表现等多元信息，综合判定最佳免疫治疗持续时间；同时设计“生物标志物分层”策略，实现对免疫治疗持续时间的动态化、精确化指导。
